# Isolation and characterization of putative functional long terminal repeat retrotransposons in the *Pyrus* genome

**DOI:** 10.1186/s13100-016-0058-8

**Published:** 2016-01-15

**Authors:** Shuang Jiang, Danying Cai, Yongwang Sun, Yuanwen Teng

**Affiliations:** Department of Horticulture, Zhejiang University, Hangzhou, Zhejiang 310058 China; Forest & Fruit Tree Institute, Shanghai Academy of Agricultural Sciences, Shanghai, 201403 China; Institute of Horticulture, Zhejiang Academy of Agricultural Sciences, Hangzhou, Zhejiang 310021 China; The Key Laboratory of Horticultural Plant Growth, Development and Quality Improvement, The Ministry of Agriculture of China, Hangzhou, Zhejiang 310058 China; Zhejiang Provincial Key Laboratory of Horticultural Plant Integrative Biology, Hangzhou, Zhejiang 310058 China

**Keywords:** Retrotransposons, Insertion time, Distribution, Genetic diversity, *Pyrus*

## Abstract

**Background:**

Long terminal repeat (LTR)-retrotransposons constitute 42.4 % of the genome of the ‘Suli’ pear (*Pyrus pyrifolia* white pear group), implying that retrotransposons have played important roles in *Pyrus* evolution. Therefore, further analysis of retrotransposons will enhance our understanding of the evolutionary history of *Pyrus*.

**Results:**

We identified 1836 LTR-retrotransposons in the ‘Suli’ pear genome, of which 440 LTR-retrotransposons were predicted to contain at least two of three gene models (*gag*, integrase and reverse transcriptase). Because these were most likely to be functional transposons, we focused our analyses on this set of 440. Most of the LTR-retrotransposons were estimated to have inserted into the genome less than 2.5 million years ago. Sequence analysis showed that the reverse transcriptase component of the identified LTR-retrotransposons was highly heterogeneous. Analyses of transcripts assembled from RNA-Seq databases of two cultivars of *Pyrus* species showed that LTR-retrotransposons were expressed in the buds and fruit of *Pyrus*. A total of 734 coding sequences in the ‘Suli’ genome were disrupted by the identified LTR-retrotransposons. Five high-copy-number LTR-retrotransposon families were identified in *Pyrus*. These families were rarely found in the genomes of *Malus* and *Prunus*, but were distributed extensively in *Pyrus* and abundance varied between species.

**Conclusions:**

We identified potentially functional, full-length LTR-retrotransposons with three gene models in the ‘Suli’ genome. The analysis of RNA-seq data demonstrated that these retrotransposons are expressed in the organs of pears. The differential copy number of LTR-retrotransposon families between *Pyrus* species suggests that the transposition of retrotransposons is an important evolutionary force driving the genetic divergence of species within the genus.

**Electronic supplementary material:**

The online version of this article (doi:10.1186/s13100-016-0058-8) contains supplementary material, which is available to authorized users.

## Background

Repetitive sequences make up a large proportion of plant genomes. Among repetitive sequences are transposable elements [1, 2], which are broken into two main classes according to their transposition intermediate: Class I retrotransposons transpose via an RNA intermediate by a “copy and paste” mechanism; and Class II transposons transpose via a DNA intermediate by a “cut and paste” mechanism [2]. LTR-retrotransposons are Class I retrotransposons that have been found in all plant species investigated to date [2–4]. These retrotransposons are flanked by LTRs and undergo replicative transposition; thus, their copy numbers increase and occupy a large portion of the genome, especially in higher plants [5–7]. For example, retrotransposons make up more than 50 % of the maize and wheat genomes [8, 9]. Active LTR-retrotransposons increase the size of plant genomes. In *Oryza australiensis*, a wild relative of rice, transposition of retrotransposons led to a rapid two-fold increase in genome size during the last 3 million years [10], suggesting that rapid amplification of LTR-retrotransposons has played a major evolutionary role in genome expansion. Environmental stress and demethylation have been hypothesized to activate retrotransposons and induce duplication events in the genome [11–13]. The retrotransposons isolated from plants appear to be young—less than 5 million years old [14]. Therefore, pathways must exist for the removal of retrotransposons. The rice genome has lost a large number of retrotransposons, corresponding to a rapid reduction in genome size [15].

Retrotransposons can insert within or near transcriptionally active regions and can cause mutations by disrupting genes, altering gene expression levels, or by driving genomic rearrangements [16, 17]. Recent evidence indicated that a retrotransposon inserted into a *myb*-related gene was associated with pigmentation loss in grape [18]. In blood orange, insertion of a retrotransposon upstream of an anthocyanin biosynthesis-related gene caused color formation in its fruit to become cold-dependent [19]. Retrotransposons display extreme sequence diversity, and there are thousands or even tens of thousands of different retrotransposon families in plants [2, 5]. An autonomous retrotransposon is composed of two nearly sister LTR sequences flanked by target site duplications of usually 4–6 bp [1]. The internal region is usually composed of two open reading frames required for replication (in some cases, LTR retrotransposons possess one unique open reading frame, such as *Tnt1*, *Tto1*, or *Tos17*): the *pol* gene encodes products with the enzymatic functions of a protease (PR), reverse transcriptase (RT) and integrase (INT); and the *gag* gene encodes structural proteins involved in the maturation and packaging of retrotransposon RNA. Conserved sequence motifs, for example, the primer-binding site and the polypurine tract are also essential for retrotransposon replication. LTR-retrotransposons can be subdivided into the Ty1-*copia* and the Ty3-*gypsy* groups based on the order of the domains encoded within *pol* genes. The order in the Ty3-*gypsy* group is PR-RT-INT, and that in the Ty1-*copia* group is PR-INT-RT [2].

The *Pyrus* L. (pear) is believed to have originated in the Tertiary period in the mountainous regions of western and southwestern China [20]. According to its original distribution area, *Pyrus* can be divided geographically into two groups: the occidental pear group and the oriental pear group [21]. The major species of oriental pear are native to China [22]. The oriental pear group contains wild pea pears and cultivated species with large fruit. Their evolutionary history is still controversial [23]. Recently, the whole genome of *P. pyrifolia* Chinese white pear ‘Suli’ was sequenced. The assembled *P. pyrifolia* genome consists of 2103 scaffolds with an N50 of 540.8 kb, totaling 512.0 Mb with 194× coverage. Sequencing and assembly revealed that much of the *P. pyrifolia* genome is retrotransposon-derived [24]; 16.9 and 25.5 % of the genome was reported to be *copia* and *gypsy* retrotransposons, respectively. A large number of retrotransposons were also found in other species in the Rosaceae family. For example, retrotransposons accounted for 37.6 and 18.6 % of the genomes of *Malus* and *Prunus* species, respectively [25, 26]. Jiang et al. (2015) reported that the retrotransposon *Ppcr1* was inserted in many loci in the genomes of cultivated *Pyrus* species, but only in a few loci in the genomes of wild *Pyrus* species [27]. This suggested that retrotransposons might play a major role in species evolution. Therefore, research on retrotransposons in *Pyrus* species will be helpful to understand the evolutionary history of *Pyrus*. Yin et al. (2014) reported that LTR retrotransposons in the *Pyrus* genome have complex structures [28], and that frequent recombination events followed by transposition of retrotransposons may have played a critical role in the evolution of *Pyrus* genomes. However, their study did not focus on the various retrotransposon families in *Pyrus* and their inner structural domains, nor did it involve the copy number of retrotransposon families in different *Pyrus* species.

In this study, we predicted the LTR-retrotransposons present in the ‘Suli’ genome, and annotated all LTR-retrotransposons with three inner functional domains (RT, INT and GAG) to identify putative functional LTR-retrotransposons. LTR-retrotransposons in the ‘Suli’ genome [24] were extremely divergent [27, 28], which made it difficult to analyze every predicted LTR-retrotransposon. Therefore, we focused on conserved LTR-retrotransposon families with a high copy number in ‘Suli’ genome, and investigated the distribution of these families in different *Pyrus* species and other closely related species to evaluate the roles of LTR-retrotransposon replication and mutation in the evolution of the *Pyrus* genome.

## Results

### Annotation and structure of LTR-retrotransposons in the ‘Suli’ genome

In previous study, a total of 1836 putative full-length LTR-retrotransposons were identified in the ‘Suli’ genome by LTRharvest. To determine which of these were most likely to be functional, we searched all identified LTR-retrotransposons for the conserved protein domains GAG, INT, and RT. A total of 440 putative LTR-retrotransposons (24.0 %) contained at least two domains and were analyzed further. Their positions in the ‘Suli’ genome and annotation information are listed in Additional file 1: Table S1 and Additional file 2, respectively. According to the order of the RT and INT domains, 373 and 67 retrotransposons belonged to the *copia* and *gypsy* groups, respectively (Table 1). *Copia*-type retrotransposons (average length, 5448 bp) were significantly shorter than *gypsy*-type retrotransposons (average length, 10,742 bp) (*p* < 0.01 by *t*-Test). The average LTR length of *copia* and *gypsy* retrotransposons was 374 and 542 bp, respectively.

**Table 1:** Characteristics of *copia* and *gypsy* putative full-length retrotransposons with more than two gene models identified in *Pyrus* genome

Type	Number	Length (nt) ± SE	5′ LTR length (nt) ± SE	3′ LTR length (nt) ± SE
*Copia*	373	5448.4 ± 1526.5	374.1 ± 138.9	374.9 ± 139.5
*Gypsy*	67	10742.0 ± 2823.7	542.4 ± 259.6	539.5 ± 259.6
*t*-Test		**	**	**

**means significant difference at the *p* < 0.01 level (*t*-Test)

**Fig. 1:**
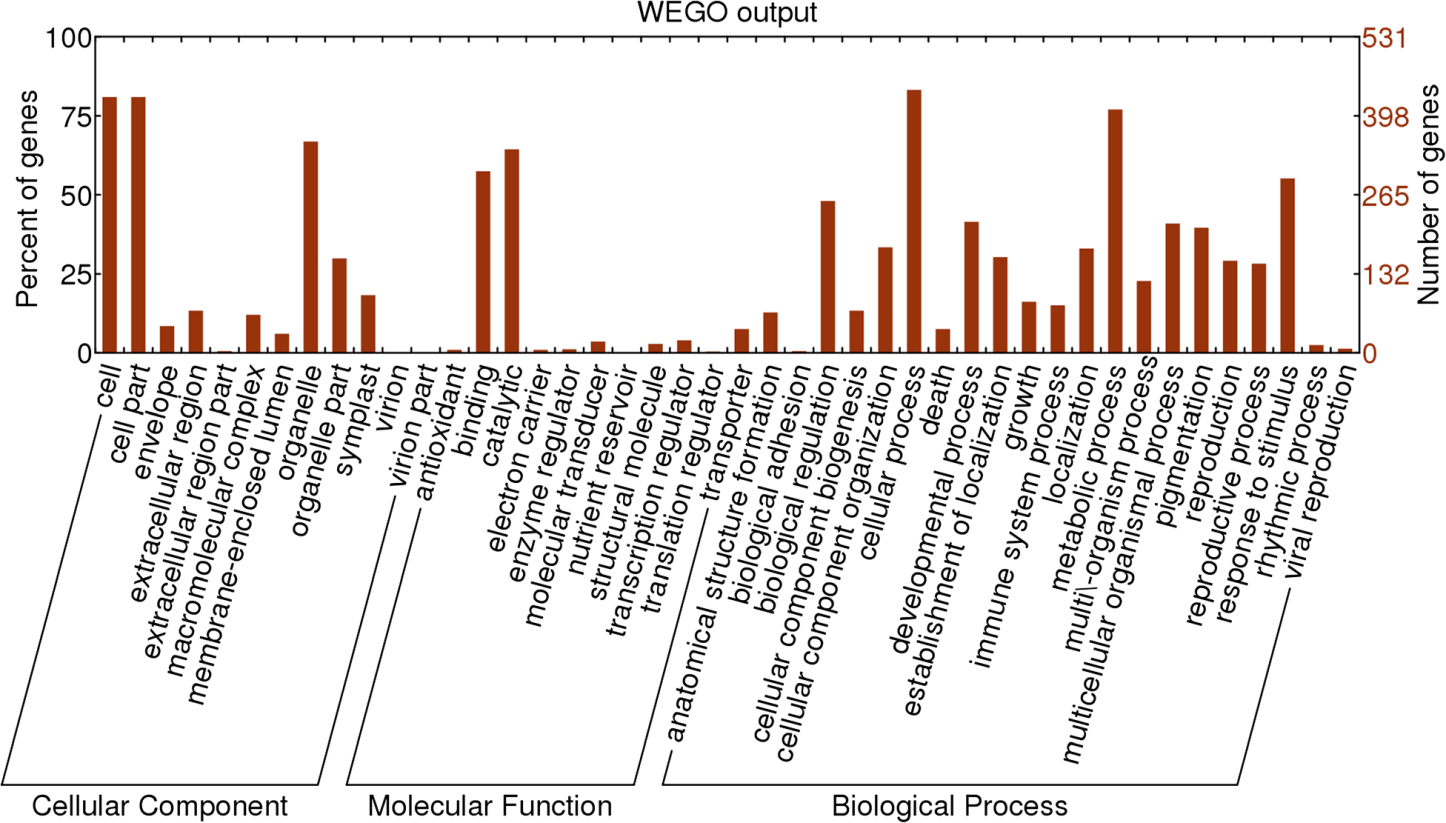
Histogram of gene ontology classifications of sequences disrupted by isolated retrotransposons. Unigenes correspond to three main categories: biological process, cellular component, and molecular function

Transposable elements can affect gene expression by disrupting functional genes or by inserting into the upstream or downstream regulatory regions of genes. We used BLAST to align our 440 conserved domain-containing LTR-retrotransposons to annotated introns in the ‘Suli’ genome, and used the Blast2GO annotation tool to assign probable gene ontology (GO) terms. A total of 734 genes aligned to LTR-retrotransposons, suggesting that they were disrupted. Of these, 531 unigenes could be annotated using GO. The unigenes were categorized into three main GO categories: biological process, cellular component, and molecular function (Fig. 1). These putatively disrupted genes were annotated using the NCBI nr database and listed in Additional file 3: Table S2. To further analyze putative retrotransposon-associated gene sequences, we searched 10,000-bp genome regions flanked by the predicted retrotransposons. A total of 2536 sequences were found, of which 1922 unigenes could be annotated using GO (data not shown).

To group the identified retrotransposons into families, we used each identified retrotransposon to conduct BLASTN searches against the whole dataset of 440 LTR-retrotransposons (coverage: 80 % and e-value: 10^−5^). In this initial effort, we identified five LTR-retrotransposon families with high-copy numbers, which we investigated further (Table 2). BLASTN searches against the Repbase database were conducted to identify conserved repetitive elements in these five families. Similar sequences identified in Repbase and reference sequences in the *Pyrus* genome are listed in Table 2. The PFAM database has many gene models related to LTR-retrotransposons. In this study, three genes (*gag*, reverse transcriptase, and integrase) were predicted to be present in high copy numbers, while the other two genes (aspartic protease and RNase H) were infrequently identified in *Pyrus* using the present gene models. Based on the predictions of three gene models, we described the structure of the five LTR-retrotransposon families isolated from *Pyrus* (Table 2, Additional file 4: Figure S1).

**Table 2:** LTR retrotransposon families investigated in this study

Family	Size (kb)	Copy number/total retrotransposons	Type	Ref Seq	ID of similar sequence in Repbase
Family I	5141	29/373	*copia*	AJSU01007348.1 (8605–13,745 bp)	Copia-24_PX
Family II	5355	15/373	*copia*	AJSU01000113.1 (27,402–32,756 bp)	Copia-106_Mad
Family III	6482	20/373	*copia*	AJSU01017137.1 (16,748–23,229 bp)	Copia-90_Mad
Family IV	5123	14/373	*copia*	AJSU01025615.1 (15,180–10,058 bp)	Copia-53_Mad
Family V	5670	5/67	*gypsy*	AJSU01016963.1 (42,874–37,205 bp)	Gypsy-5_Mad

### Putative insertion time of LTR-retrotransposons

**Fig. 2:**
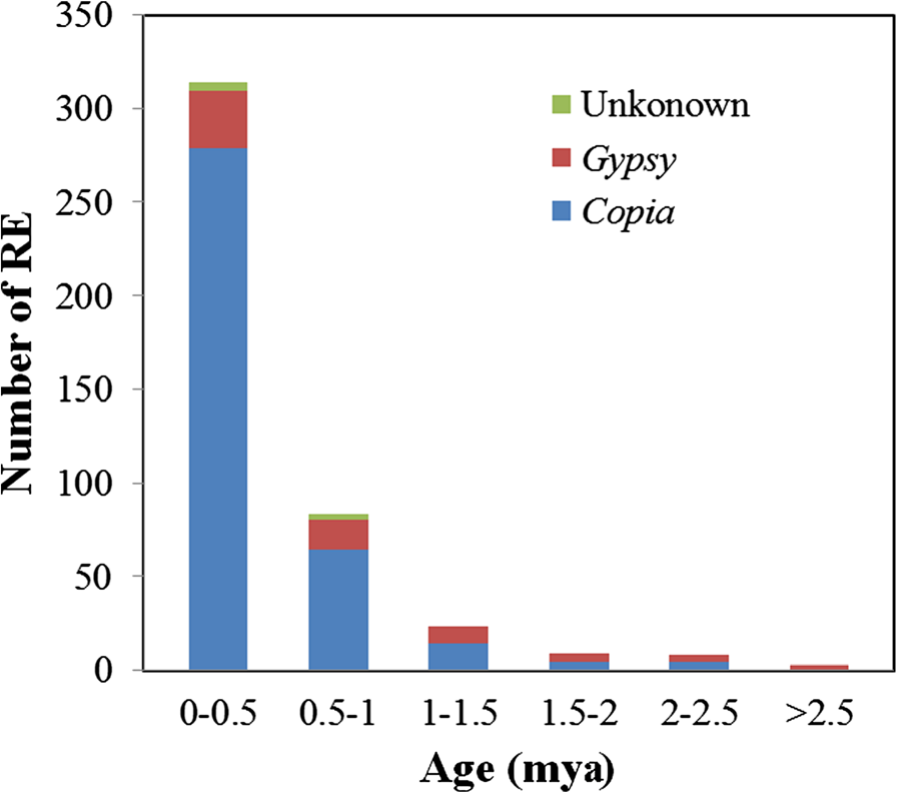
Insertion time of 440 retrotransposons identified in ‘Suli’ genome

The insertion time of LTR-retrotransposons was estimated by analyzing the divergence of sister LTRs. We used the molecular clock rate of 1.3 × 10^−8^ substitutions per site per year [29]. The insertion time can only be considered as a rough estimate, and only large differences should be considered significant. The divergence between sister LTRs ranged from 0 to 0.076, representing a maximum insertion time of 2.93 MYA. The predicted mean insertion time of the 440 LTR-retrotransposons analyzed in this study was 0.42 MYA. The predicted mean insertion time of *copia*-type LTR-retrotransposons was 0.35 MYA, significantly shorter than the predicted insertion time of 0.81 MYA (*p* < 0.01 by *t*-Test) for *gypsy*-type LTR-retrotransposons. Most of the retrotransposons were estimated to have inserted into the genome during the last 2.5 million years (Fig. 2). The peak of retrotransposon mobilization was observed at 0–0.5 MYA, indicating that our predicted retrotransposons were inserted relatively recently.

The mean insertion time of each member of the five LTR-retrotransposon families was estimated to be within the last 1 million years (Additional file 5: Figure S2). One member from Family I was inserted 1.75 MYA. In Families II, III, IV, and V, some members did not show LTR variations, indicating that they were inserted into the genome recently.

### Phylogenetic relationships among isolated LTR-retrotransposons

**Fig. 3:**
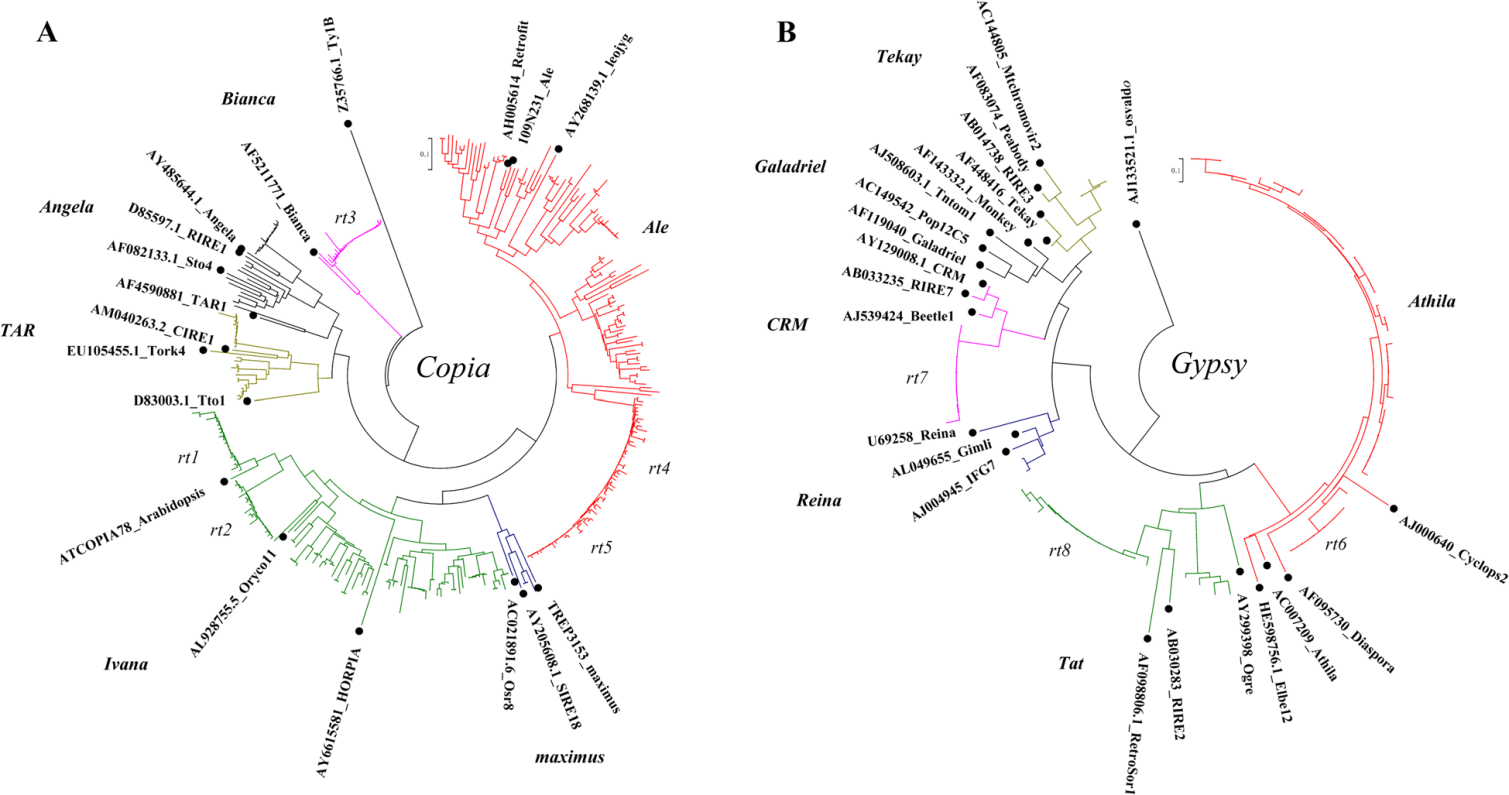
Phylogenetic relationship of RT sequences based on translated nucleotide sequence from identified retrotransposons in ‘Suli’ genome. **a** Phylogenetic tree of *copia*-type RT sequences based on 329 RTs in ‘Suli’ pear and 17 identified RTs. **b** Phylogenetic tree of *gypsy*-type RT sequences based on 67 RTs in ‘Suli’ pear and 22 identified RTs

The LTR-retrotransposons showed wide variations in their full-length sequences and could not be clustered. To evaluate the relationship among predicted LTR-retrotransposons, we used the neighbor-joining method to cluster the translated nucleotide sequences of reverse transcriptase (*rt*) in our identified LTR-retrotransposons with known TE families (Fig. 3). Both translated *copia*- and *gypsy*-type RT sequences clustered into many groups (Fig. 3). Although there was wide divergence among RT sequences, five and three conserved clades of RT sequences were identified among *copia* and *gypsy* retrotransposons, respectively. The average divergence of untranslated *copia-* and *gypsy*-type *rt* sequences was 0.64 and 0.55, respectively, indicating high heterogeneities among *rt* sequences (data not shown). Five *rt* sequences from each conserved clade of *copia* retrotransposons were aligned (Additional file 6: Figure S3), and the sequence divergence ranged from 0.068 to 0.691. *rt4* and *rt5* were similar. For the *gypsy* retrotransposons, the sequences of *rt6*, *rt7*, and *rt8* were aligned (Additional file 6: Figure S3), and their sequence divergences were 0.775, 0.898, and 0.98, respectively.

### Transcriptional analysis of LTR-retrotransposons in various organs in *Pyrus*

**Fig. 4:**
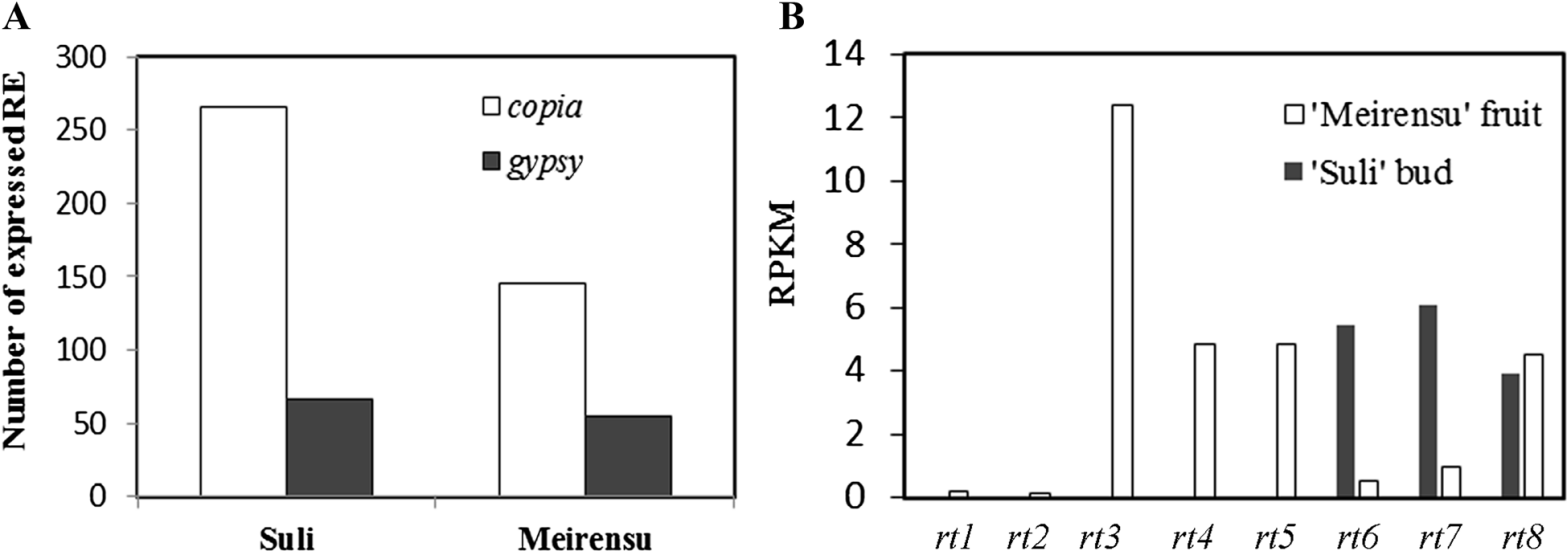
Frequency of transcriptionally active retrotransposons present in two *Pyrus* transcriptomes. **a** Number of expressed retrotransposons. **b** The value of reads per kilobase of gene model per million reads for eight types of RT

Two transcriptomes assembled from RNA-Seq datasets were used in this study. A total of 116,182 sequences (62.6 Mb) assembled from 19,878,957 reads collected from buds of ‘Suli’ (SRX147917) and 36,495 sequences (15.8 Mb) assembled from 452,428,795 reads collected from fruit of *P. pyrifolia* ‘Meirensu’ (SAMN03857509-SAMN03857515) were aligned using BLAST to the 440 LTR-retrotransposons that we identified. LTR-retrotransposons were transcriptionally active in both the fruit and bud (Fig. 4). A total of 266 *copia*-type and 66 *gypsy*-type LTR-retrotransposons aligned with transcripts from the bud of ‘Suli’ and 146 *copia*-type and 55 *gypsy*-type LTR-retrotransposons aligned with transcripts from the fruit of ‘Meirensu’, indicating that these retrotransposons were expressed (Fig. 4). Because the normalized expression values of individual retrotransposons were very low (data not shown), we only showed the reads per kilobase of gene model per million reads values of eight RT families (*rt*1–*rt*8). In fruit of ‘Meirensu’, the high transcription level of *rt*3 were represented.

### Distribution of LTR-retrotransposon families among *Pyrus* species

To determine the exact copy number of LTR-retrotransposons, we used the reverse transcriptase gene model to search the database of protein sequences translated from ‘Suli’ genome data with Hmmer3.0. A total of 8144 *copia*-type RTs and 3748 *gypsy*-type RTs were identified. According to the average length of *copia* and *gypsy* retrotransposons (Table 1), *copia* and *gypsy* retrotransposons accounted for 8.8 % (42.3 Mb) and 8.0 % (38.4 Mb) of the genome, respectively.

**Fig. 5:**
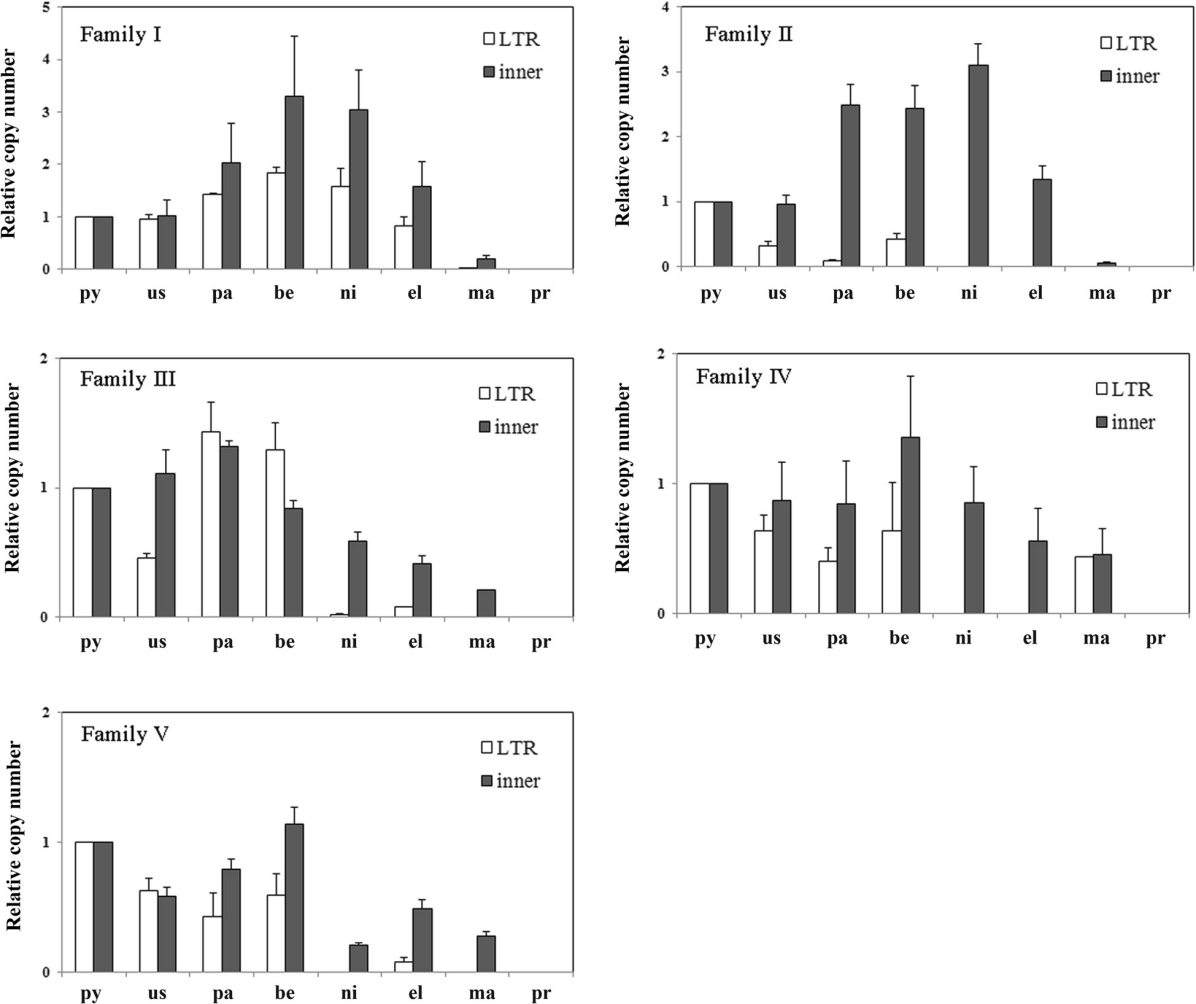
Distribution of retrotransposon families in *Pyrus*, *Malus*, and *Prunus*. py, *P. pyrifolia* white pear ‘Suli’; us, *P. ussuriensis* ‘Balixiang’; pa, *P. pashia*; be, *P. betulaefolia*; ni, *P. nivalis*; el, *P. elaeagrifolia*; ma, *Malus* × *domestica* ‘Fuji’ and pr, *Prunus persica* ‘Hujingmilu’

The distribution of LTR-retrotransposon families was estimated in different *Pyrus* species and related species. *Pyrus* species exhibited little variation in genome size (Additional file 7: Table S3). We could not calculate the exact copy number of retrotransposons in *Pyrus*, but the relative copy number could be measured by real-time quantitative PCR (Q-PCR). Analyses of the LTR and inner sequences of five LTR-retrotransposon families showed that all LTR-retrotransposon families were present in all *Pyrus* species and *Malus* × *domestica*, but not in *Prunus persica* (Fig. 5). Families I and II were found infrequently in *Malus* genomes and two cultivated pear species (*P. pyrifolia* and *P. ussuriensis*), but they were abundant in the genomes of three wild pear species (*P. pashia*, *P. betulaefolia*, and *P. nivalis*). Interestingly, families II, III, and IV in *P. elaeagrifolia* and *P. nivalis*, exhibited increased copy number of the inner sequence relative to LTRs of retrotransposons. The copy numbers of family III and V retrotransposons were higher in oriental pears than in occidental pears.

## Discussion

### Distribution and duplication of *copia* and *gypsy* retrotransposons in *Pyrus*

Recent evidence showed that a large proportion of retrotransposons were non-functional because of mutations in their protein-coding domains [30]. In this study, we identified predicted LTR-retrotransposons in the ‘Suli’ genome, and focused on LTR-retrotransposons that had the highest likelihood of being functional based on the presence of annotated inner protein domains. Previously, we identified 1836 retrotransposons by running LTR-harvest based on two nearly sister LTR flanking sequences and some conserved sequence motifs [27]. However, the current study showed that only 440 retrotransposons had at least two inner protein domains. This finding suggests that there are very few full-length retrotransposons, and even fewer potentially functional LTR-retrotransposons in the *Pyrus* genome.

In a previous study, *copia* and *gypsy* retrotransposons were reported to account for 16.9 and 25.5 % (ratio, 0.66) of the genome of the ‘Suli’ pear, respectively [24]. However, in the present study, *copia* and *gypsy* retrotransposons were estimated to account for 8.8 and 8.0 % (ratio, 1.1) of the genome of the ‘Suli’ pear, respectively, based on RT gene models. Our predictions focused on the existence of *rt* gene in LTR retrotransposons, which is essential for retrotransposon transposition. Therefore, the retrotransposons predicted in this study may be functional, suggesting that at least 60 % of retrotransposons in the ‘Suli’ pear genome lack *rt* genes, and are therefore unable to replicate. Previous studies have established that lacking *rt* genes causes many LTR retrotransposons to be non-functional entities within host genomes [30].

### High heterogeneity of LTR-retrotransposons in ‘Suli’ genome

The sequences and sequence length differed significantly among the full-length LTR-retrotransposons from the ‘Suli’ genome. We analyzed *rt* sequences to evaluate the diversity of retrotransposons. Our data showed that the average divergence of *rt* sequences in *copia-* and *gypsy-*family retrotransposons was 0.64 and 0.55, respectively. These findings indicate that the *rt* sequences from pear are highly heterogeneous (Fig. 3), like those in rice [31], strawberry [32] and masson pine [33]. There could be several reasons for the observed high sequence heterogeneity. First, gene mutation is the major cause of heterogeneity. In recent reports, many retrotransposons were existed in the genome for a long time [31, 34]. In this study, some retrotransposons were predicted to exist before the speciation of *Pyrus* and *Malus* based on sequence divergence (Fig. 5). The long period since the first retrotransposon insertion events is one potential source of variation. Both active and non-functional retrotransposons would have accumulated mutations over time, giving rise to a highly heterogeneous population [1]. Second, all transposons are integrated into chromosomal DNA. Therefore, mutated retrotransposon sequences, carrying mainly nonsense mutations are heritable, permitting a high degree of heterogeneity of retrotransposons between generations. Third, the high divergence between *rt* sequences of the LTR-retrotransposons we identified suggests a complex origin. For example, the divergence between *rt6* and *rt7* and between *rt6* and *rt8* was 0.898 and 0.98, respectively, suggesting that the origin of these related retrotransposons was complex, rather than from a single source. High sequence heterogeneity is the main obstacle that makes it difficult to classify retrotransposons as *copia*- or *gypsy*-types. In this study, we identified five related families of LTR-retrotransposons (Table 2). The members of each family showed high similarity and were strongly conserved, suggesting that these families have duplicated many times in recent years.

### The insertion time of LTR-retrotransposon in ‘Suli’ genome

The divergence of sister LTR sequences was used to estimate the insertion time of retrotransposons. When an LTR-retrotransposon is inserted into the genome, the similarity of LTR sequences is 100 %. As time passes, mutations occur within the two LTRs, resulting in a larger genetic distance between them. In this study, only putative full-length LTR-retrotransposons were analyzed, and annotation of LTRs was performed by LTRharvest, which is known to be biased toward recent insertions of LTR-retrotransposons. Therefore, only recently inserted LTR-retrotransposons might be identified in our study. Our data showed that the majority of the retrotransposons we identified in the ‘Suli’ genome were inserted into the genome over the last 2.5 million years (Fig. 2). It was estimated that *Pyrus* and *Malus* diverged from each other between 5.4 and 21.5 MYA [24], suggesting that mobilization of these retrotransposons occurred frequently in the evolution of *Pyrus* species after the divergence of *Malus* and *Pyrus*. Within the retrotransposon families, the majority of members of families I–IV were estimated to have inserted into the genome over the last 1 million years (Additional file 5: Figure S2), confirming that these retrotransposons in *Pyrus* were inserted into the genome only recently.

### Transcription of LTR-retrotransposons in pear organs

The expression of LTR-retrotransposons is likely to be silent in plant tissue during normal development. Many retrotransposons are expressed and transposed in protoplasts [35], and some are activated by abiotic stresses [11, 36]. In our study, the isolated retrotransposon sequences were aligned against the assembled transcriptomes of ‘Suli’ pear buds (SRX147917) and ‘Meirensu’ pear fruit (SAMN03857509-SAMN03857515) using BLAST. The expression of retrotransposons was detected in the fruit and buds of *Pyrus* cultivars (Fig. 4), which suggested that retrotransposons are expressed in *Pyrus* organs under normal conditions of growth and development. The expression of retrotransposons is advantageous for replication of retrotransposons, and retrotransposon transposition commonly results in mutation [18, 19]. In pear fruit and buds, retrotransposons showed transcriptional activity, which could increase their copy number in the genome. The mutations in buds and seeds could be transmitted to the next generation. The high rates of retrotransposon expression and transposition may contribute to the large proportion of retrotransposons in the *Pyrus* genome (as high as 42.4 %) [24].

### Genetic diversity of LTR-retrotransposons in *Pyrus* and other close-related genera

Multiple studies support the hypothesis that retrotransposons might be associated with the evolution of plant genomes [7, 15]. In *Pyrus*, we identified 440 full-length LTR-retrotransposons that differed significantly from each other (Fig. 3). Five high copy-number retrotransposon families (four from the *copia* group and one from the *gypsy* group) were identified to further analyze the diversity of retrotransposons in *Pyrus* and other closely related genera. All five LTR-retrotransposon families were detected in six *Pyrus* species (Fig. 5), among which *P. betulaefolia* and *P. pashia* are believed to be the ancestral species in the genus *Pyrus* [23, 37]. The detection of a large number of retrotransposons indicates that these retrotransposons have widely existed in pear species for a long time. However, these five LTR-retrotransposon families were rare in *Malus*, and absent from *Prunus* (Fig. 5), indicating that they were duplicated and increased their copy number in *Pyrus* genomes after the differentiation of *Pyrus* and *Malus*. Both *Malus* and *Prunus* genomes contain a large number of retrotransposons [25], which are likely descended from different families than those found in *Pyrus.* These results suggest that the evolution of retrotransposons has varied among the different genera in the Rosaceae family.

Retrotransposons have played a major role in changing the size of genomes by either increasing genome size [10] or promoting rapid genomic DNA loss [15]. In *Pyrus*, the genome size does not vary greatly among species (Additional file 7: Table S3). Therefore, we can estimate the relative copy number of retrotransposon families in different *Pyrus* species. Our result shows that the copy number of retrotransposon families differs in *Pyrus* species. For example, *P. nivalis*, *P. pashia* and *P. betulaefolia* have a higher copy number of family I and II LTR-retrotransposons than *P. pyrifolia*, *P. ussuriensis*, and *P. elaeagrifolia*. In addition, *P. nivalis* has a low copy number of family III and V, implying these families were lost in *P. nivalis* evolution*.* The changes in the number of retrotransposon families might cause genetic divergence in *Pyrus* species. In *P. betulaefolia*, all five LTR-retrotransposon families showed high copy numbers in the genome, indicating that this species has a larger proportion of retrotransposons in the genome than other *Pyrus* species. *Pyrus nivalis* and *P. elaeagrifolia* have a low copy number of the LTR regions of retrotransposons in families II, III and IV. The LTR region of these families might be lost and formed solo LTRs, or this region might have mutated. We inferred that the retrotransposon families have mutated and duplicated highly during the evolution of *Pyrus*.

## Conclusions

We predicted 440 full-length LTR-retrotransposons from the ‘Suli’ pear genome, and annotated three inner protein domain sequences (GAG, INT, and RT) in retrotransposons, suggesting that the isolated retrotransposons might be functional. The analysis of three RNA-Seq databases of buds and fruit in different *Pyrus* cultivars showed retrotransposons were still active in pear organs. The isolated retrotransposons were highly heterogeneous. They had existed in *Pyrus* species for a long time, but have rapidly expanded during the last 2.5 million years after the divergence of *Malus* and *Pyrus*. Our results showed that the copy number of retrotransposon families varied among *Pyrus* species. To our knowledge, this is the first investigation of genetic variation of retrotransposons within the genus *Pyrus*. These findings support that retrotransposon transposition is an important evolutionary force driving the genetic divergence of species within the genus *Pyrus*.

## Methods

### Plant materials and DNA extraction

The plant materials used in this study consisted of six *Pyrus* accessions (two oriental cultivars: *P. pyrifolia* Chinese white pear ‘Suli’ and *P. ussuriensis* ‘Balixiang’, two oriental wild species: *P. pashia* and *P. betulaefolia*, and two occidental wild species: *P. nivalis* and *P. elaeagrifolia*), *Malus* × *domestica* ‘Fuji’, and *Prunus persica* ‘Hujingmilu’. Genomic DNA was extracted from the young leaves of each specimen using the modified CTAB protocol described by JJ Doyle and JL Doyle [38] The precise concentration of DNA was detected using DNAQF-1KT (Sigma, St Louis, MO, USA). The DNA concentration of each sample was diluted to 1 ng · μl^−1^, and 1 μl was used as a template for real-time quantitative PCR analysis.

### Identification and annotation of LTR-retrotransposons

In a previous study, 1836 full-length LTR-retrotransposons were mined from the whole-genome data of *Pyrus* (AJSU00000000) [27]. The details of each retrotransposon were obtained from the output of LTRharvest. All retrotransposons were translated into proteins in all six possible reading frames using an in-house Perl script. All of the *copia* and *gypsy* gene models were downloaded from the PFAM database (*gag*, PF03732; integrase, PF00665; reverse transcriptase, PF00078 and PF07727). Each gene model was used to search all of the proteins translated from retrotransposons with Hmmer3.0 software. To describe the genes around retrotransposons, 10,000 bp upstream and downstream of each LTR-retrotransposon were annotated with the BLAST algorithm using Blast2GO, and the results were visualized using the WEGO tool [39]. In the ‘Suli’ genome, a total of 42,812 coding genes were identified [24], and we searched gene introns isolated from the *Pyrus* genome to detect genes that were disrupted by retrotransposons.

### Phylogenetic analyzes

According to the position of *rt* in the Hmmer3.0 results, we calculated the start and end of the *rt* sequences in the assembled ‘Suli’ genome. An in-house Perl script was used to extract nucleotide sequences from the whole-genome data, and translated them to amino acid sequences. The amino acid sequences of RT in *copia* and *gypsy* retrotransposons were aligned with known TE families, including *Maximus*, *Ivana*, *Ale*, *Angela*, *TAR*, *Bianca* in *copia* elements and *Athila*, *Tat*, *Tekay*, *CRM*, *Reina*, *Galadriel* in *gypsy* elements separately using ClustalW, and a neighbor-joining tree was constructed based on their genetic distance using Mega 5.2 software [40].

### Estimation of insertion time of full-length LTR-retrotransposons

Bioperl scripts were used to automate the process of estimating the time of retrotransposon insertion. The two LTRs of each isolated retrotransposon were first aligned using ClustalW 2.0 [41], and genetic divergence between the two LTRs was estimated using the baseml module of PAML4 [42]. The insertion time (*T*) was estimated for each LTR-retrotransposon using the formula T = *k* / 2*r*, where *k* is the divergence between two LTRs and *r* is the substitution rate of 1.3 × 10^−8^ substitutions/site/year [29].

### Estimation of LTR-retrotransposon copy number by Q-PCR

Q-PCR was used to estimate the copy number of retrotransposons in the genome [43]. We aligned five retrotransposon families with the *Malus* and *Prunus* genomes using BLAST, and designed Q-PCR primers (Additional file 8: Table S4) in the conserved region of LTR and inner domain using Primer 3 software (http://primer3.ut.ee/). The reaction solution (total volume, 20 μl) consisted of 10.0 μl SYBR Premix Ex Taq (Takara, Shiga, Japan), 0.4 μl each primer (10 μM), 1 μl DNA (1 ng · μl^−1^), and 7.2 μl double distilled water. The reaction, performed on a LightCycler 1.5 instrument (Roche, Mannheim, Germany), started with a preliminary step of 95 °C for 30 s followed by 40 cycles of 95 °C for 5 s and 60 °C for 20 s. A template-free control for each primer pair was set for each run. Three biological replicates were used and three measurements were performed on each replicate. The relative copy number of each sample was calculated using the Ct value [43].

### Transcriptional analysis of retrotransposons in various organs/tissues of *Pyrus*

The Illumina RNA-Seq data from two samples were downloaded from NCBI. Data from buds (*P. pyrifolia* CWP ‘Suli’, SRX147917) and fruits (*P. pyrifolia* ‘Meirensu’, SAMN03857509-SAMN03857515) were analyzed to identify the transcriptional patterns of isolated retrotransposons. Raw sequence data in fastq format were filtered to remove reads containing adaptors, reads with more than 5 % unknown nucleotides, and low-quality reads with more than 20 % bases with a quality value of ≤10. Only clean reads were used in the following analyzes. Transcriptome *de novo* assembly was carried out using the short-read assembly program Trinity [44]. Two transcript databases were obtained for BLAST searches, and the isolated LTR-retrotransposons were used to identify the activity of each retrotransposon.

## Additional files

Additional file 1: Table S1. Annotation of 440 isolated LTR retrotransposons. (XLSX 88 kb) Additional file 2:
**The nucleotide sequences of 440 isolated LTR retrotransposons analyzed in the study.** (FASTA 2783 kb) Additional file 3: Table S2. List of disrupted genes. (XLSX 40 kb) Additional file 4: Figure S1. Structure of five retrotransposon families in *Pyrus*. (TIF 729 kb) Additional file 5: Figure S2. Insertion times of members of retrotransposon families I–V. (TIF 43 kb) Additional file 6: Figure S3. Alignment of five *rt* sequences from each conserved clade of *copia* retrotransposons and three *rt* sequences from each conserved clade of *gypsy* retrotransposons. (TIF 264 kb) Additional file 7: Table S3. Genome size of *Pyrus* species and related species. (DOCX 19 kb) Additional file 8: Table S4. Primers used in this study. (XLSX 10 kb)

## Abbreviations

LTR: Long terminal repeat
*rt*: Reverse transcriptase
