# Supplementary figures and images for: Isolation and characterization of putative functional long terminal repeat retrotransposons in the Pyrus genome
Source: Mob DNA. 2016 Jan 15;7:1. doi: 10.1186/s13100-016-0058-8 (PMC4715297; doi:10.1186/s13100-016-0058-8)

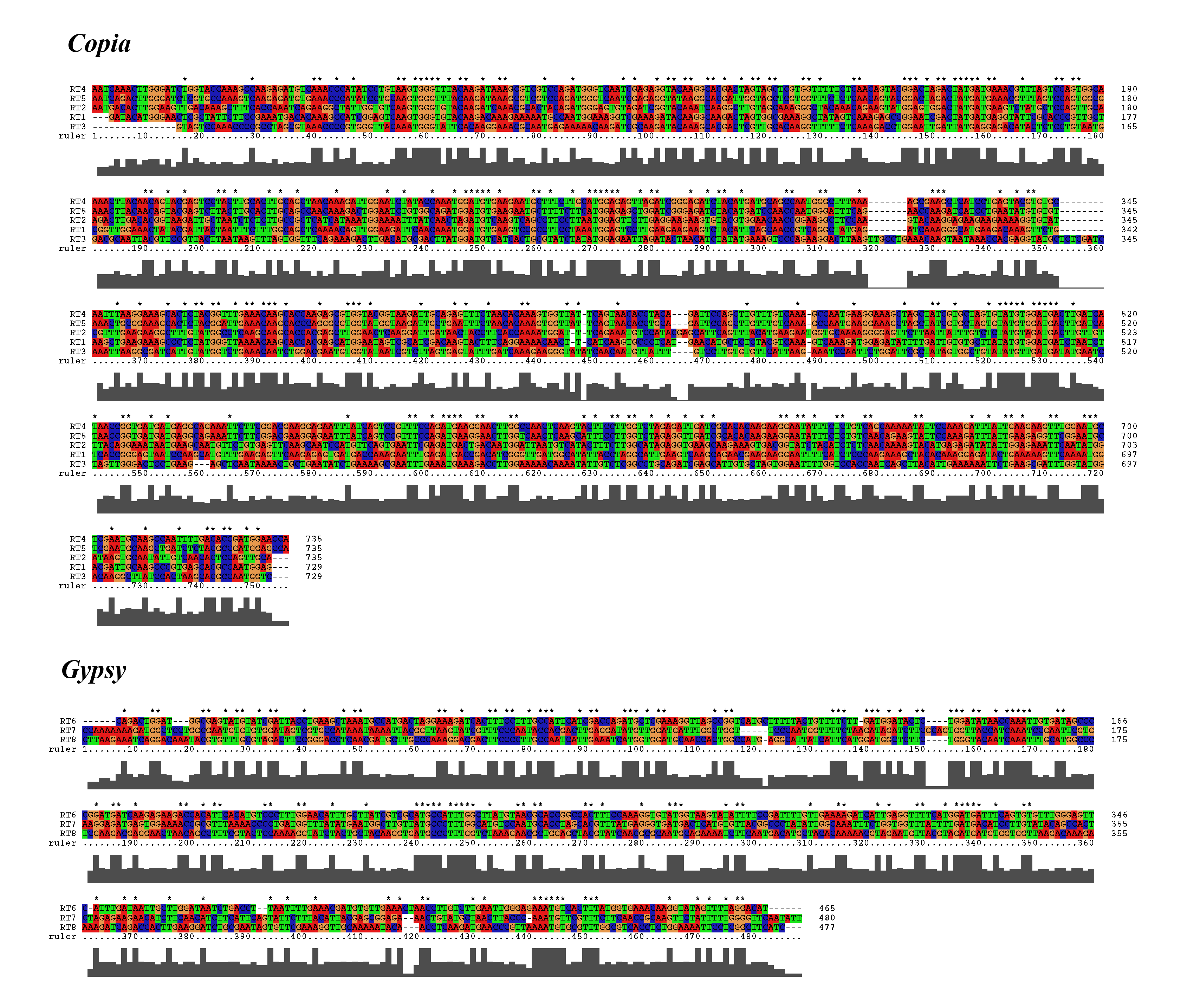

Supplement: Additional file 4: Figure S1. — Structure of five retrotransposon families in Pyrus. (TIF 729 kb) [file 13100_2016_58_MOESM4_ESM.tif]

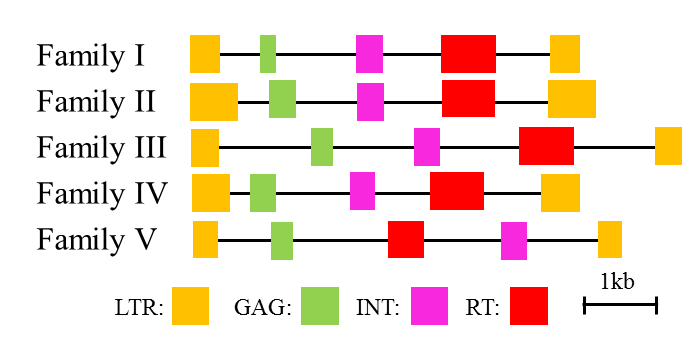

Supplement: Additional file 5: Figure S2. — Insertion times of members of retrotransposon families I–V. (TIF 43 kb) [file 13100_2016_58_MOESM5_ESM.tif]

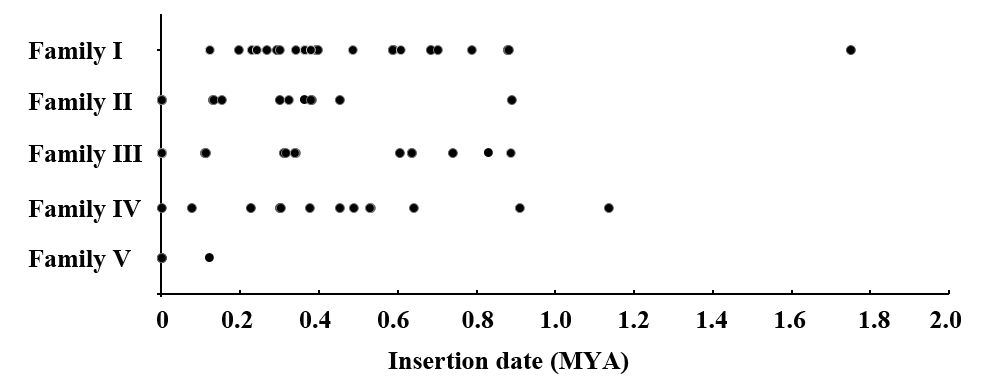

Supplement: Additional file 6: Figure S3. — Alignment of five rt sequences from each conserved clade of copia retrotransposons and three rt sequences from each conserved clade of gypsy retrotransposons. (TIF 264 kb) [file 13100_2016_58_MOESM6_ESM.tif]
