# Supplementary material for: Isolation and characterization of putative functional long terminal repeat retrotransposons in the Pyrus genome
Source: Mob DNA. 2016 Jan 15;7:1. doi: 10.1186/s13100-016-0058-8 (PMC4715297; doi:10.1186/s13100-016-0058-8)
Supplement: Additional file 7: Table S3. — Genome size of Pyrus species and related species. (DOCX 19 kb) [file 13100_2016_58_MOESM7_ESM.docx]

Table S3 Genome size of *Pyrus* species and related species.

| Species name | 2C DNA (pg, mean ± SD) | Genome size* (MB) | Reference |
| --- | --- | --- | --- |
| *Pyrus elaeagrifolia* | 1.15 ± 0.01 | 562.35 ± 4.89 | [[1](#_ENREF_1)] |
| *Pyrus communis* | 1.03/1.11 | 503.67/542.79 | [[2](#_ENREF_2)] |
| *Pyrus calleryana* | 1.26 ± 0.29 | 616.14 ± 141.81 | [[3](#_ENREF_3)] |
| *Pyrus pyrifolia* white pear group | / | 512.0 | [[4](#_ENREF_4)] |
| *Malus* × *domestica* | 1.54–1.65 | 753-807 | [[2](#_ENREF_2)] |
| *Prunus persica* | 0.54/0.55 | 264/269 | [[2](#_ENREF_2)] |

*1 pg = 489 Mb [[5](#_ENREF_5)].

1. Jedrzejczyk I, Sliwinska E: **Leaves and seeds as materials for flow cytometric estimation of the genome size of 11 rosaceae woody species containing DNA-Staining inhibitors**. *Journal of Botany* 2010:Article ID 930895.

2. Arumuganathan K, Earle ED: **Nuclear DNA content of some important plant species**. *Plant molecular biology reporter* 1991, **9(3)**:208-218.

3. Dickson EE, Arumuganathan K, Kresovich S, Doyle JJ: **Nuclear-DNA Content Variation within the Rosaceae**. *American Journal Of Botany* 1992, **79**(9):1081-1086.

4. Wu J, Wang Z, Shi Z, Zhang S, Ming R, Zhu S, Khan MA, Tao S, Korban SS, Wang H *et al*: **The genome of the pear (*Pyrus bretschneideri* Rehd.)**. *Genome research* 2013, **23**(2):396-408.

5. Dolezel J, Bartos J, Voglmayr H, Greilhuber J: **Nuclear DNA content and genome size of trout and human**. *Cytom Part A* 2003, **51A**(2):127-128.
